# Assessing healthcare provider knowledge of human trafficking

**DOI:** 10.1371/journal.pone.0264338

**Published:** 2022-03-09

**Authors:** Nicole E. Exeni McAmis, Angela C. Mirabella, Elizabeth M. McCarthy, Cara A. Cama, Miklos C. Fogarasi, Listy A. Thomas, Richard S. Feinn, Ivelisse Rivera-Godreau

**Affiliations:** 1 Stanford University, Palo Alto, CA, United States of America; 2 Stanford Health Care, Palo Alto, CA, United States of America; 3 Frank H. Netter MD School of Medicine, Quinnipiac University, North Haven, CT, United States of America; 4 St. Luke’s University Health Network, Bethlehem, PA, United States of America; Centers for Disease Control and Prevention, UNITED STATES

## Abstract

**Background and objectives:**

Human trafficking is a significant problem in which healthcare workers are in a unique position to intervene. This study sought to determine the self-reported knowledge levels of healthcare providers most likely to come in direct contact with victims of human trafficking.

**Methods:**

An anonymous survey assessing self-reported knowledge of human trafficking was developed and distributed online. Demographic information and questions pertaining to training and knowledge of trafficking in a healthcare setting were asked. The primary outcomes were descriptive statistics and secondary outcomes were comparisons among demographic groups. Qualitative methodology via content analysis was implemented on an open-ended question.

**Results:**

The 6,603 respondents represented all regions of the country. Medical, nursing, and physician assistant students comprised 23% of the sample, while 40% were either physicians, fellows, or residents. Less than half the respondents (42%) have received formal training in human trafficking, while an overwhelming majority (93%) believe they would benefit by such training. Overall, respondents thought their level of knowledge of trafficking was average to below average (mean = 2.64 on a 5-point scale). There were significant differences in knowledge of trafficking by age group (p < .001), region (p < .001), and educational training level (p < .001). 949 respondents (14.4%) provided free-text comments that further described their opinions.

**Conclusion:**

Most respondents stated they have not received training but felt they would benefit from it. There were significant differences between demographic groups. Further innovation is needed to design a universally appropriate curriculum on human trafficking that is accessible to all healthcare providers as well as mandatory training programs for healthcare institutions.

## Introduction

Human trafficking is a profound violation of human rights and is a local, national, and global health problem. Victims are reduced to objects for commerce, leading to a $150 billion-dollar industry representing the second largest source of income for organized crime [1, 2]. While human trafficking is kept clandestine, a recent report showed that over 40.3 million people worldwide are victims with over 70% being women and girls and one in four victims being children under the age of 18 [3, 4]. While once assumed to be a mostly international problem with 5.4 victims of modern slavery for every thousand people, the US has an estimated 1.3 victims per every thousand people [4, 5]. In response, the *Federal Strategic Action Plan on Services for Victims of Human Trafficking* was created to increase the coordination, collaboration, and capacity of federal agencies in the fight against human trafficking [6].

Healthcare providers are one of the few professionals who are likely to interact with victims of human trafficking [1, 7]. Multiple studies have found that up to 88% of victims had come into contact with the healthcare system while being trafficked [8–11]. These victims are most likely to seek medical care from emergency departments (63.3%), Planned Parenthood clinics (29.6%), private practices (22.5%), urgent care clinics (21.4%), women’s health clinics (19.4%), and neighborhood clinics (19.4%) [10].

Trafficking survivors noted that there was often a delay between the onset of their injury or illness and their interaction with a healthcare provider [9]. Thus, providers in these settings are in an opportune position, as this may be the only time that a victim can engage in a one-on-one discussion with a trusted professional [12]. Providers can offer both important medical and psychological care for these victims as they suffer from a wide range of health risks due to their circumstances and experiences [12–15]. Common concerns that prompt contact with the healthcare system infectious diseases, trauma or injury from physical violence, sexual abuse, malnutrition, dental disease, posttraumatic stress disorder (PTSD), anxiety, depression, or substance use disorders [10, 12–18]. Studies have found that virtually every body system has been affected. In many cases, these symptoms are vague and not necessarily directly related to the trafficking experience, but likely a consequence of the lifestyle they have been forced to practice [19].

According to the U.S. Department of Health and Human Services, healthcare providers should focus on providing support and services for four main needs: immediate medical need, mental health assistance, income support, and legal status for international victims [18]. Unfortunately, many victims may not be recognized for several reasons including lack of knowledge by healthcare providers, the control of the victim’s visit by a trafficker, the fear or shame the victim may experience, or social or cultural alienation [9, 12]. One study found that 84% of patients did not disclose victimization due to shame and 76.9% of patients were unidentified due to lack of inquiries by healthcare providers [20]. The stigma associated with sex work, which is just one aspect of human trafficking, adds a layer of difficulty to the identification of victims and often precludes patients from engaging in treatment. Cultural idiosyncrasies may also be a barrier for identification as victims may repress psychological issues secondary to their own beliefs regarding seeking care [21]. Another aspect of this situation is that once human trafficking is identified, ongoing follow-up can become challenging due to high rates of disengagement, loss of contact, etc. Therefore, it is important to apply a trauma and survivor-centered approach, as this could create an effective line of communication that can result in a successful engagement with the healthcare system [22]. The psychological trauma experienced by these victims often cause barriers that can be broken through the use of trauma-informed practices, ultimately creating a safe environment [22]. Unfortunately, identified victims may not receive the needed referrals or assistance due to lack of response protocols, safe disposition, and internal resources [12].

For healthcare providers to fully assist victims of human trafficking, further awareness, knowledge, and training is needed to help identify and assess vulnerable patients. Healthcare providers are becoming increasingly aware of human trafficking victims in healthcare settings; however, they continue to state that they have insufficient training in order to recognize these individuals [23]. Additionally, healthcare providers often lack knowledge and implementation of trauma-informed practices, especially in physical care that may mimic the experience of abuse [24, 25]. Unfortunately, it was also found that due to healthcare provider inexperience, survivors of human trafficking had negative experiences in healthcare settings including inappropriate physical exams, breaches of confidentiality, and disbelief by provider of trafficking status [26]. Further innovation is needed to not only raise awareness of the problem of human trafficking, but to also create educational programs that incorporate items such as trauma-informed and evidence-based training, cultural awareness, and survivor-informed interview questions [27]. This is incredibly important as healthcare providers are often the only professionals that can speak privately to victims and where victim’s statements can be later used to seek prosecution against traffickers [10].

Healthcare providers have the opportunity to interact with victims and disrupt the cycle of abuse [7, 9, 10, 12]. They can screen, identify, intervene, and make a plan of action to help victims. However, there are limited studies detailing the amount of knowledge that healthcare providers have regarding this problem. Therefore, a survey study was conducted to determine the self-reported knowledge levels of healthcare providers who are more likely to be in direct contact with a potential human trafficking victim.

## Materials and methods

### Survey development

A subjective survey of self-reported knowledge of human trafficking among healthcare providers was developed through an iterative process, including discussions with colleagues and members of the study team. The survey was reviewed and refined for content validity by two currently practicing physicians (co-authors L.T., I.G.).

The survey (data in S1 Table) included questions on demographic characteristics, prior human trafficking training, need for such training, and ten items pertaining to knowledge of human trafficking in a healthcare setting. Participants were asked “How would you rank your knowledge?” to each of these ten items on a five-point Likert scale (very low, below average, average, above average, very high) (Table 1). An open-ended question at the end of the survey offered a chance for additional commentary.

**Table 1:** Survey question #7.

Item
Role in identifying and responding to human trafficking
Indicators or red flags of human trafficking
Practices where victims typically present
Appropriate questions to ask to identify a victim
Common chief complaints
Common chronic health problems (PMHx)
Documentation in an EMR when suspecting a victim
Local and/or national support
Local and/or national policies
Knowledge of appropriate referrals to recommend to a victim

This table demonstrates the 10 knowledge items assessed regarding human trafficking in the survey.

The primary method of accessing and completing the survey was via an online survey software (Qualtrics XM). The survey was only accessible for completion once per respondent. Prior to initiating the survey, the Quinnipiac University Institutional Review Board (IRB) approved the study under exempt category 2(i) from the Code of Federal Regulations (45 CFR 46.104.D) and assigned it project number 00420.

### Participants

Survey participants included a national sample of EMTs, fellows, medical assistants, medical students, nurses, nurse practitioners, nursing students, paramedics, physicians, physician assistants, physician assistant students, residents, and social workers. No identifying data from the participants was recorded.

### Survey administration

The survey was distributed nationally via email and medical online forums. Emails were sent to physicians, physician assistants, and nurses spanning a variety of specialties from all academic medical centers, PA programs, and nursing schools across the country. Emails were also sent to the board of directors as well as deans of medical schools, PA programs, and nursing schools across the country. Directors of EMS organizations, nursing organizations, PA organizations, and physician organizations were also contacted via email directly. Emails were sent to all potential participants twice in order to ensure that those who would like to respond had the opportunity. Online forum requests to complete the survey were submitted to the following: Society of General Internal Medicine (SGIM); American College of Emergency Physicians (ACEP); Emergency Medicine Residents’ Association (EMRA); Society of Hospital Medicine (SHM); and American Academy of Family Physicians (AAFP). Forum requests were placed twice, 4 months apart, in order to ensure that those who would like to respond had the opportunity. All email invitations and medical online forum invitations included eligibility criteria for participants and the statement of waived consent.

### Statistical and qualitative analysis

Frequencies and percentages were used for descriptive statistics. Pearson chi-square was used to test for associations between demographic and categorical variables. The ten items regarding knowledge of trafficking in a healthcare setting were averaged to create an overall knowledge score. Factor analysis of the items resulted in the first eigenvalue equaling 7.3 and the second eigenvalue 0.7, supporting a single factor. Further, reliability analysis of the 10 items resulted in excellent internal consistency (Cronbach’s alpha = 0.96). The Friedman nonparametric test for related samples was used to compare the 10 Likert items and a one-way ANOVA with Bonferroni post-hoc comparisons was used to compare demographics groups on the created knowledge score. Analyses were performed with SPSS v26.

Responses to an open-ended question (“additional commentary”) were reviewed using qualitative methodology via content analysis [28]. Four authors (N.M., A.M., E.M., C.C.) individually reviewed all responses to identify key content organized into categories.

## Results

Table 2 shows the demographic characteristics of the 6,603 respondents. Approximately half (52%) of the respondents were under age 40 and two-thirds (66%) were female. All regions of the country were well represented and a small percentage (0.8%) were from outside the United States. The respondents included physicians (33.7%), medical students (13.6%), nurses (9.8%), PA students (7.0%), paramedics (6.1%), social workers (5.7%), residents (5.5%), physician assistants (5.1%), EMTs (4.9%), nurse practitioners (4.3%), nursing students (2.2%), medical assistants (1.3%), and fellows (0.7%).

**Table 2:** Demographic characteristics.

Characteristic	Frequency	Percentage (%)
**Age Group**		
21–30	1892	28.7
31–40	1579	23.9
41–50	1268	19.2
51–60	988	15.0
61–70	723	10.9
71–80	138	2.1
81–90	15	0.2
**Gender**		
Female	4370	66.2
Male	2206	33.4
Non-binary	27	0.4
**Region**		
Northeast	1435	21.7
Midwest	1429	21.6
South	2465	37.3
West	1223	18.5
Outside US	50	0.8
**Level of Training**		
EMT	325	4.9
Fellow	46	0.7
Medical Assistant	88	1.3
Medical Student	901	13.6
Nurse	650	9.8
Nurse Practitioner	281	4.3
Nursing Student	142	2.2
Paramedic	406	6.1
Physician	2223	33.7
Physician Assistant	337	5.1
PA Student	464	7.0
Resident	362	5.5
Social Worker	378	5.7

This table demonstrates the demographic information of survey participants.

As shown in Table 3, less than half of respondents (42%) have received training in human trafficking, while an overwhelming majority (93%) believe they would benefit from such training. There were significant differences in receiving training by age group (p < .001), region (p < .001), and training (p < .001). The age group with the highest percentage of training was 51–60 years old (48.6%) and it was progressively lower as the age groups became younger and older. Only 34.1% and 20.0% of 71–80 and 81–90 respectively were trained, while just 33.4% of the 21–30 age group received training. Training was highest in the Midwest (53.5%) and lowest (30.0%) for respondents outside of the United States. Medical assistants had the lowest percentage of training (13.6%) followed by nursing students (14.8%), while social workers had the highest percentage (60.1%) followed by nurse practitioners (56.6%). Every nursing student (100%) and most medical students (98.6%) and physician assistant students (98.3%) felt human trafficking training would be beneficial. Physicians gave the lowest affirmative response (87.8%) to this question.

**Table 3:** Responses to human trafficking training questions.

Question	Frequency	Percentage (%)
**Received Training in Human Trafficking**		
No	3819	57.8
Yes	2784	42.2
**Would Benefit from Human Trafficking Training**		
No	435	6.6
Yes	6168	93.4

This table demonstrates the statistics of participants who have previously received training in human trafficking as well as those who felt they would benefit from training.

**Fig 1:**
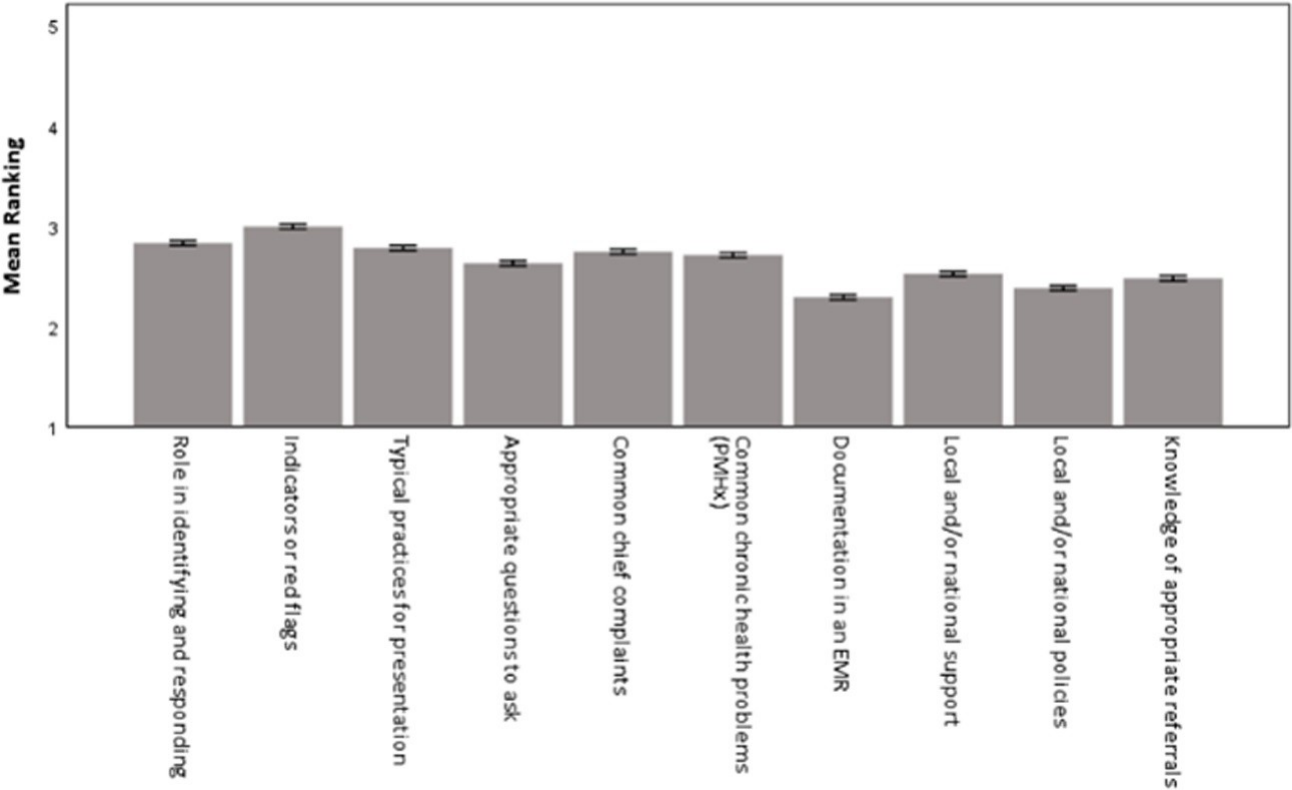
Mean (95% CI) response for each knowledge item. This figure demonstrates the mean response for each of the 10 knowledge items listed in Table 1.

Fig 1 shows the mean response for each of the ten knowledge items. There was a significant difference in the ranking between items (p < .001). Almost all pairwise comparisons were significant even after Bonferroni adjustment. Strikingly, the mean rank for every item was below the scale midpoint of 3 (average), suggesting suboptimal levels of knowledge. The highest mean ranks were for items ‘indictors or red flags of human trafficking’ (M = 2.99) and ‘role in identifying and responding to human trafficking’ (M = 2.83) and the lowest mean ranks were for ‘documentation in an EMR’ (M = 2.29) and ‘local and/or national policies’ (M = 2.38). The mean knowledge score, comprised of the ten items, for the entire sample of 6603 respondents was 2.64 (SD = 0.85).

**Fig 2:**
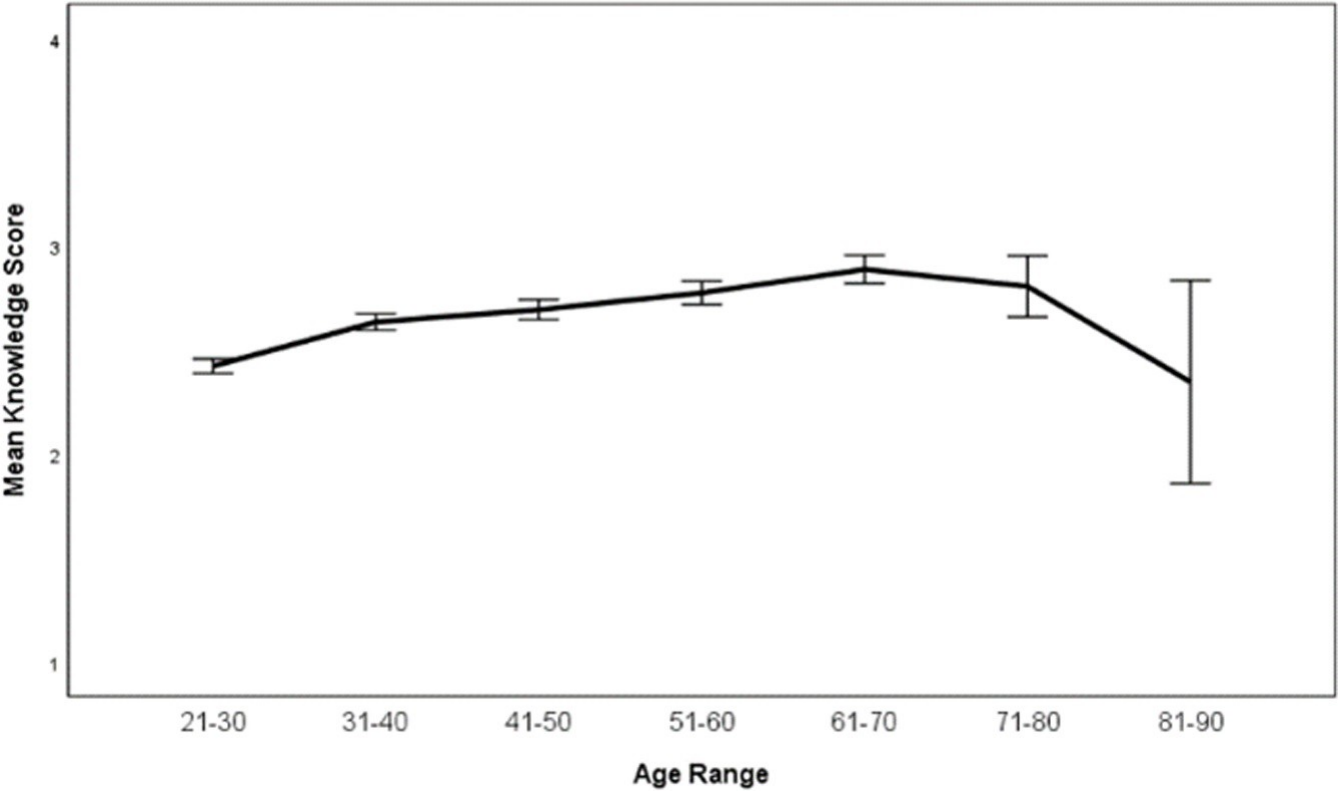
Mean (95%CI) knowledge score by age group. This figure demonstrates the mean knowledge score on a Likert scale from 1–5 dependent on age group.

There were significant differences between demographic groups on the knowledge score. Age groups significantly differed (p < .001) but there was not a linear trend across ages (p = .647). However, there was a significant quadratic trend (p < .001) with age group 61–70 having the highest mean (M = 2.89) and age groups 81–90 and 21–30 the lowest means (M = 2.35 and 2.42 respectively) (Fig 2).

**Fig 3:**
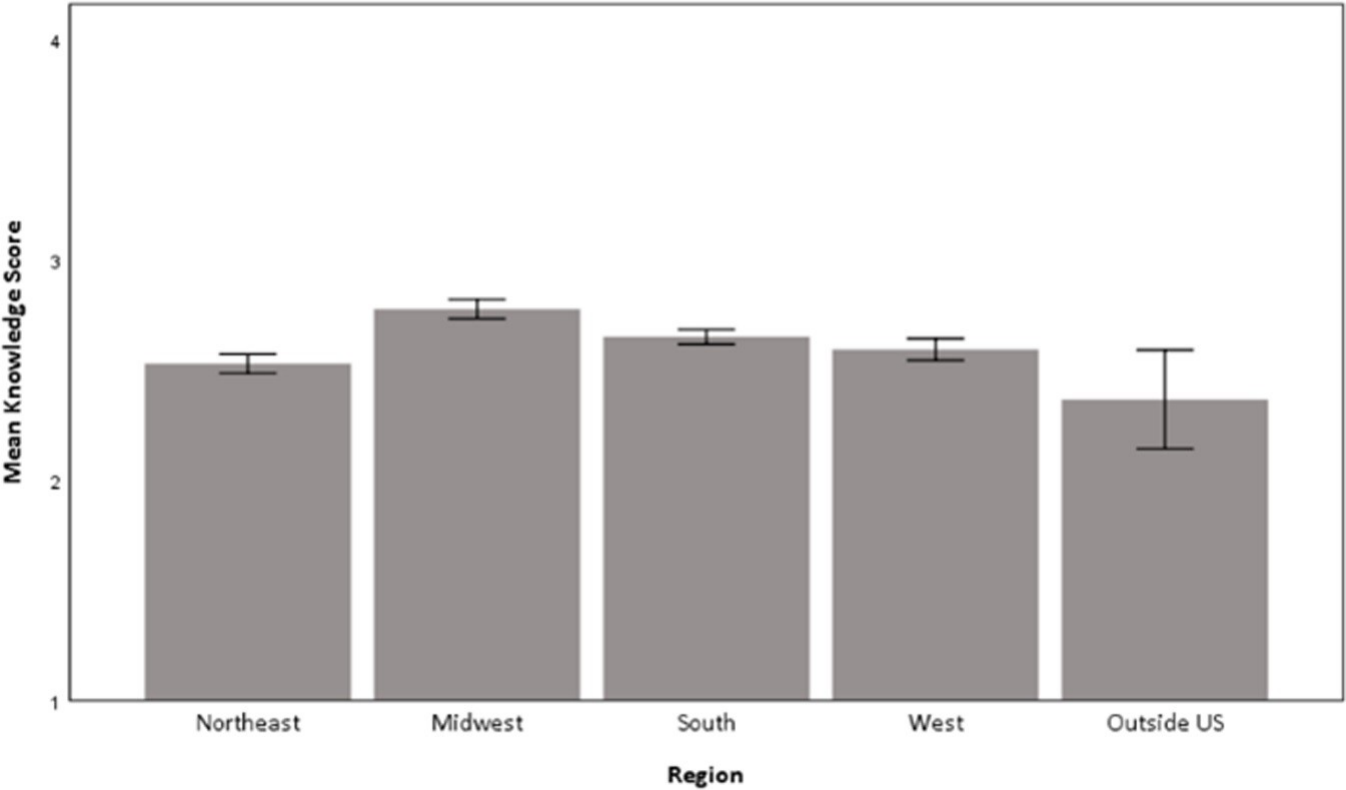
Mean (95%CI) knowledge score by region. This figure demonstrates the mean knowledge score on a Likert scale from 1–5 dependent on region.

There was no significant difference by gender (p = .227) but there was by region (p < .001). Respondents from the Midwest had the highest mean score (M = 2.78) while those outside of the US (M = 2.37) and from the Northeast (M = 2.53) had the lowest means (Fig 3).

**Fig 4:**
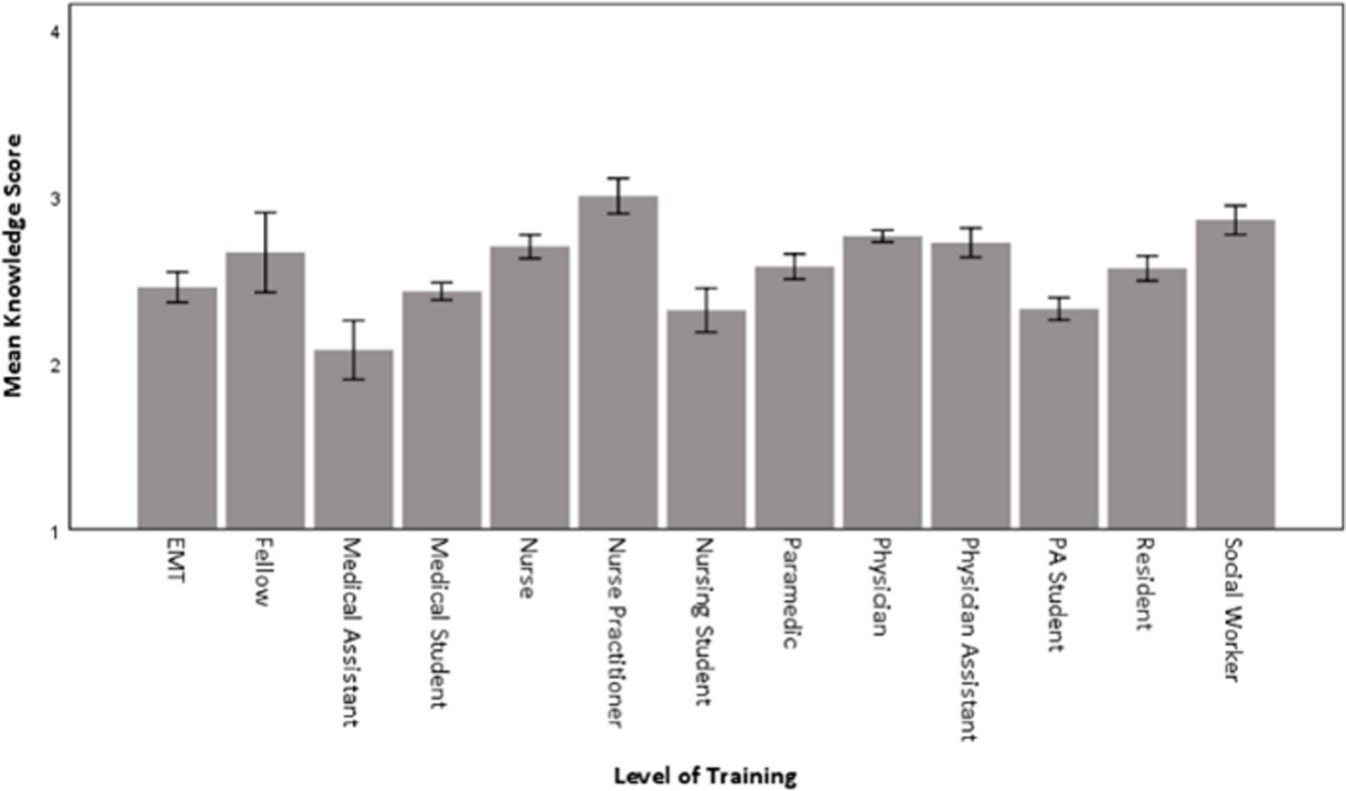
Mean (95%CI) knowledge score by level of training. This figure demonstrates the mean knowledge score on a Likert scale from 1–5 dependent on level of training.

There was a significant difference by level of training (p < .001). As seen in Fig 4, the highest means were from nurse practitioners (M = 3.01) and social workers (M = 2.86), and the lowest means from medical assistants (M = 2.08) followed by nursing students (M = 2.32), physician assistant students (M = 2.33), and medical students (M = 2.43).

### Opinions: Free-text responses

949 respondents (14.4%) provided detailed responses to the final open-ended question. Most responders commented on details of the survey and on perceived need of training in their own field. Finally, some noted where their knowledge was obtained, which states have mandated training, or recommendations for future training.

#### Raising awareness

Positive comments expressed thanks for raising awareness on a topic where knowledge is lacking in multiple fields. Others noted that this survey helped them realize their lack of or need to refresh their knowledge regarding this topic. For example, respondents commented:

“This survey made me aware of my lack of knowledge in this subject. This truly sparked a feeling of obligation in me as a future health care provider to become informed about the subject.”

“Never occurred to me that I might need training in this area, but taking the survey made it very clear to me that I do.”

“I have treated many traumatized and abused patient in public hospital and clinic settings. Think trafficking victims might have similar presentations, but realize in taking your survey, have no definite knowledge of how to specifically identify and care for these patients.”

“These questions have made me look at myself and the knowledge that I have about this topic.”

“This survey has made me feel inadequate, but has motivated me to take initiative about learning more about this field.”

“I didn’t realize I know nothing about human trafficking until today and it would be great information as a provider.”

#### Prior knowledge and training

Another major topic was how respondents obtained prior knowledge/training. Participation in hospital lectures, CME training, church seminars, Truckers Against Trafficking (TAT) training, American College of Physicians (ACP) training, SOAR training, HEAL Trafficking training, and Learn to Identify and Fight Trafficking (LIFT) training were among the top free-text responses. Others obtained training or knowledge from working in locations where human trafficking is prevalent:

“Our facility serves a very vulnerable population with a high prevalence of trafficked individuals. We have had educational sessions/programs, have in-dept services (that can dedicate their time to secondary screening/managing cases initially screened/identified by MD/RN) and we participate in regional multi-agency activities related to trafficking (including provide medical care for individuals brought in related to law enforcement raids targeting trafficking).”

Respondents mentioned if their knowledge was from further certification, such as Sexual Assault Nurse Examiners (SANE) certification or Forensic Nurse certification. It was found that human trafficking education is required by some states like Michigan, Florida, and Texas to maintain licensing for nurses, nurse practitioners, and social workers. Of note, while the training does not need to be in-person, it does need to contain specific content as identified by legislation, however the content does vary by state. There was a large number of comments made by Emergency Medicine, OB/GYN, and Psychiatric physicians and nurses noting that they had extensive knowledge given their specialty of choice. Also, United States military healthcare providers are trained yearly in combating human trafficking. Personal experiences with victims also serve as educational experiences. The following comments illustrate this:

“I have properly identified human trafficking victims and provided a safe-haven for these victims…We were able to get her back home…safely, with her family, after weeks of work on her case.”

“In addition to being an EMS provider and a 911 dispatcher, I am also the parent of children who were sex trafficked through their daycare.”

Many respondents mentioned how this self-assessment survey made them realize that while they have previously received training, they are still lacking the knowledge needed to help victims.

“I have served on a volunteer committee for human trafficking and even I am nowhere close to feeling comfortable with treating patients involved in human trafficking yet. I think this goes to show just how important more training on this topic is.”

“I had a patient that I suspect was involved in human trafficking but only realized months later because of an unrelated comment by an attending about common trafficker techniques. Whether or not she was involved in human trafficking, not recognizing it or looking into it more will haunt me the rest of my career.”

#### Specific specialties

Various responders did not feel that human trafficking training is relevant to their current practice setting due to either their patient population or location. Amongst those who commented, those working in sports medicine, geriatrics, oncology, neonatology, anesthesiology, and infectious disease did not feel the need to be trained given their specialty.

“Training would be useful only for physicians who are likely to deal with patients who are victims of human trafficking.”

“My work setting is not one where a victim of [human trafficking] is at all likely to present. Primary care and inpatient settings would benefit more.”

Some felt they may simply be uninformed about the issue to recognize the need:

“I don’t believe I encounter this regularly in my area of practice, but then, I suppose I might not know if I did due to lack of training.”

Others viewed training as unnecessary if they are nearly or already retired. Although many noted that they do not interact with human trafficking victims, they still felt that knowledge in identifying victims is relevant for all healthcare providers.

#### Ideal training and request for further training

A subset of comments featured survey responders’ efforts to design an ideal training program on human trafficking. A majority centered around a need for training that results in lasting knowledge, including actionable items and concrete skills building. It was noted that many current trainings focus on why human trafficking is an important issue; however, there was little information that could be used in practice.

“I think this is research is…much needed. As practitioners, in our training, we very quickly are taught general clues to look for, but no methodical way of identifying victims. I think more formal training would be extremely beneficial.”

An emphasis was placed on creating a more practical training that includes identifying red flags, how to approach potential victims, what questions to use to screen, common chief complaints or specific health issues, how to document the encounter in the EMR, and education about resources available for victims.

“A readily available, posted community resource list would be welcome. Descriptions of how to manage the separation of the individual being trafficked from their handler, the prolonged visit, transportation to safe places, and managing the handler would be helpful part of training."

“No doubt this is crucial. Like many issues, it would benefit from practice tools and a ‘telescoped’ approach with targeted but effective ways to identify, refer to more skilled providers or resources, and tools to assist overwhelmed frontline practitioners.”

“Such a training needs to be really well planned—most trainings on these topics are actually not helpful nor do they build skills—they too often end up being knowledge sharing and making a case for why this is an important issue, but little substance. I’ve gone to trainings in the past—and while my knowledge of the topic (stats, policies) increased, there was extremely little applicable to my practice. If you design a training—you should strongly consider planning 80–90% to concrete/actionable skills building—e.g., ‘how to ask about trafficking, what questions to use to screen, how to document, chief complains/specific health issues that may be a red flag, identifying red flags, etc.).’ “

Another typical suggestion was for consistent, longitudinal training with repetition due to the complex, constantly changing facts, data, and trends on the issue.

“One-time training is not sufficient. Many people get the training, know the material, but still don’t recognize the person sitting across from them is a victim. The "Do you feel safe at home?" is a good started question, but we know it’s not enough …”

“Human trafficking is a pervasive. It can be blatant or nuanced. Although I have been trained and I currently incorporate screening for human trafficking into every patient encounter, more training is always helpful. I realize that the traffickers become increasingly clever as more is exposed about their operations.”

“Continuing education about human trafficking with current data, current stories, and resources will always be invaluable to reducing/eliminating this horrifying practice.”

Many respondents expressed a desire for easily accessible and interactive training, such as online or web-based training. One suggested a readily available phone application for easily accessible information on all aspects of human trafficking. Others suggested simulation and standardized patients to practice questioning and interacting with potential victims.

“What we really need is a live workshop with role playing on how to actually intervene in the moment when the victim is refusing help. That’s the real challenge here.”

“I think training that would especially be helpful is given a series of patients and try to decide who is a suspected victim of sex trafficking and who isn’t. That will really test our skills!”

However, some also expressed concern because institutions do not have a systematic way of identifying human trafficking victims. Others felt that this training had not been implemented in their region as human trafficking would not happen in their town, city, or area.

There were many requests for further training. Some respondents suggested mandating training in all aspects of healthcare as well as integrating training into professional graduate school curriculum and residency programs. Others mentioned that training should be tailored towards specific specialties such as Emergency Medicine and OB/GYN. Medical student, physician assistant student, and resident responders mentioned that they needed to seek out their own training because training was not provided or readily available. Furthermore, if training was provided, it was limited to once a year specifically on statistics instead of how to identify and treat victims. Unfortunately, comments were made regarding the time limitations present for learning an additional topic as well as the lack of this topic on national licensing board exams.

“Like anything, this sounds like a really important issue, but providers and providers’ time is so limited with patients so I would educate the clinicians most likely to encounter these victims (to make a significant, but efficient impact).”

“It’s all a balance of time and effort. Human trafficking is worthy of information, but it is competing against all other acute clinical conditions.”

## Discussion

In this widely distributed self-assessment survey, the majority of the over 6,600 participants from various levels of training believed that they would benefit from human trafficking training with less than 50% having previous training.

Age group, geographical region, and level of training were associated with varying knowledge levels. Participants in the age group from 61–70 were found to have the highest level of knowledge when compared to age groups above and below those numbers. Knowledge was highest in the Midwest with the South, West, and Northeast following closely behind. While there was a very small number of international respondents, it is still worth noting that those outside the United States had the lowest level of training. Amongst all the training levels, nurse practitioners were noted to have the highest knowledge level followed closely by social workers. Nursing students had the lowest percentage of training, but every nursing student believed they would benefit from training. Following closely in knowledge level were medical students and physician assistant students.

Overall, “average” was the most common responses to the knowledge questions. More responses were in the “very low” or “below average” category when compared to the “above average” or “very high” category. Across all knowledge questions, the mean rank for each question was below the scale midpoint of 3 with an average knowledge score across all respondents as 2.64. This indicates the need and potential benefit for human trafficking training across all levels of training in the healthcare field.

Another important nuance was captured by the high proportion of written short response answers in the survey (>900 comments). Qualitative analysis of the data identified previously obtained levels of training and ideas for improvement in training. The comments in this study identified the various methods that respondents used to learn about human trafficking. Many noted that training should focus on information that is actionable and concrete in order to build skills in identifying and treating victims. Another important aspect included the request for further training regardless of the form of training provided and the need for training in all levels of healthcare.

There are several limitations to this study. Information regarding the type of practice or specialty of the responding nurses, physicians, physician assistants, and social workers was not collected. Specific degrees such as doctor of nurse practitioner, counselor, mid-wife, and others were not included as options in the survey. There may have been selection bias as the participant pool was skewed towards certain subspecialties such as emergency medicine, internal medicine, family medicine, obstetrics and gynecology, and psychiatry. Additionally, further self-selection bias may have occurred when potential participants have prior interest in this issue. Those who have prior experience in human trafficking may have been more likely to respond to this self-assessment survey as well as offer support for more training on the subject. Participants were not surveyed regarding the type of previous training or how often they received this training. Also, where the participant obtained knowledge if they did not receive formal training was not assessed. The survey also did not fully define the proposed scale of “very low, below average, average, above average, and high” or define what “formal training” entails.

Identifying a need for further education is crucial to the overall advancement of identifying victims of human trafficking; however, the type of education is paramount. Despite having some respondents with prior training, the average response for knowledge was still below the midpoint level on the 5-point scale, possibly indicating the need for, not just more training but more quality training. Healthcare providers are in a unique position to serve as the first responders for victims of human trafficking. While healthcare providers have many resources available to their disposal, further steps may include the creation of a universally appropriate curriculum on human trafficking that all healthcare providers can access, mandatory training programs for all institutions, and a universal consensus on the best tools to identify victims. Such curriculum would optimally focus on indicators or red flags, common medical, physical, and psychological conditions, and health concerns of human trafficking victims, while incorporating appropriate questions and interviewing techniques needed to approach a suspected victim. Ideally training would also incorporate the perspectives of human trafficking survivors, as opposed to current training models established through perceptions of healthcare workers [23]. The implementation of training focused on the views and needs of victims and survivors help healthcare workers respond more appropriately. Incorporating training through the help of survivors has been shown to improve potential outcomes including survivor mental health, consistent reporting, recognition of barriers to care delivery, and more [23]. Training inclusive of the insights of human trafficking survivors is necessary to help respond to the unique needs of this population while providing a depth of culturally appropriate and safe care [26]. While there are few elements that have been proposed regarding effective training, many studies have found that it is paramount to include trauma-informed primary care in conjunction with mental health services [22]. Even though the implementation of trauma-informed care has been shown to be effective, its use is not currently widespread. It was found that providers who were not trained appropriately in trauma-informed care viewed this type of care as more of a barrier, further proving the need for additional implementation [29]. A curriculum incorporating the theory of trauma-informed practices in clinical practice with survivors of human trafficking could be a possible scenario. For example, the Dignity Health Methodist Hospital of Sacramento Family Medicine Residency Program has created the Human Trafficking Medical Safe Haven program model that can be easily integrated into care for survivors as well as adapted for a diverse range of clinical environments.

Furthermore, implementation of a universal training program would allow for more effective responses from the healthcare field. Human trafficking training should be included across all types of medical education, including medical school, residency programs, fellowship programs, nursing programs, PA programs, etc [30]. This type of training would allow healthcare providers to provide a neutral and safe environment conducive to strong connections with trafficking victims. Further studies may be needed to determine the format of training that would be most useful amongst different healthcare professionals. Additional studies to determine which professionals or individuals would be best equipped to create and implement such training are also needed as there is currently no research available.

## Conclusion

This self-assessment survey of over 6,600 participants from various levels of the healthcare field illustrates the overall need for enhanced training in human trafficking. Institutional and health-systems leaders can use this information to advocate for continued and improved training in human trafficking identification for all healthcare providers. Based on the literature, access to effective training models that increase knowledge in trauma-informed care practices could affect the application of these skills to identify and manage victims of human trafficking. It is also important to consider research on educational programs that can be effective and accessible to the multiple disciplines of healthcare.

## Supporting information

S1 Table Survey questions. (DOCX)
