# Supplementary material for: Assessing healthcare provider knowledge of human trafficking
Source: PLoS One. 2022 Mar 9;17(3):e0264338. doi: 10.1371/journal.pone.0264338 (PMC8906613; doi:10.1371/journal.pone.0264338)
Supplement: S1 Table — (DOCX) [file pone.0264338.s001.docx]

**S1 Table. Survey questions.**

| **#** | **Question** | **Response Options** |
| --- | --- | --- |
| **1** | Level of training | EMTs, fellows, medical assistants, medical students, nurses, nurse practitioners, nursing students, paramedics, physicians, physician assistants, physician assistant students, residents, social workers |
| **2** | Gender | Male, Female, Non-binary |
| **3** | Age Range | 21-30, 31-40, 41-50, 51-60, 61-70, 71-80, 81-90 |
| **4** | Have you received training in identifying victims of human trafficking? | Yes, No |
| **5** | Are you aware of the statistics on human trafficking? | Yes, No |
| **6** | How would you rank your knowledge in identifying a trafficked individual? | Very Low, Below Average, Average, Above Average, Very High |
| **7** | How would you rank your knowledge in the following?   - Role in identifying and responding to victims of human trafficking. - Indicators or red flags of human trafficking. - Practices where victims typically present. - Appropriate questions to ask to identify a victim. - Common chief complaints. - Common chronic health problems (PMHx). - Documentation in an EMR when suspecting a victim. - Local and/or national support. - Local and/or national policies. - Knowledge of appropriate referrals to recommend to a victim. | Very Low, Below Average, Average, Above Average, Very High |
| **8** | Do you feel that you would benefit from human trafficking training? | Yes, No |
| **9** | Additional commentary. | N/A |
